# Three-Dimensional Printing of a Hemorrhagic Cervical Cancer Model for Postgraduate Gynecological Training

**DOI:** 10.7759/cureus.950

**Published:** 2017-01-01

**Authors:** Michael Bartellas, Stephen Ryan, Gregory Doucet, Deanna Murphy, Jacqueline Turner

**Affiliations:** 1 Medicine, Memorial University of Newfoundland; 2 Faculty of Medicine, Memorial University of Newfoundland; 3 Faculty of Engineering and Applied Science, Memorial University of Newfoundland

**Keywords:** three-dimensional printing, simulation, post-graduate training, cervix, neoplasm, cancer, hybrid simulation

## Abstract

**Introduction:**

A realistic hemorrhagic cervical cancer model was three-dimensionally (3D) printed and used in a postgraduate medical simulation training session.

**Materials and methods:**

Computer-assisted design (CAD) software was the platform of choice to create and refine the cervical model. Once the prototype was finalized, another software allowed for the addition of a neoplastic mass, which included openings for bleeding from the neoplasm and cervical os. 3D printing was done using two desktop printers and three different materials. An emergency medicine simulation case was presented to obstetrics and gynecology residents who were at varying stages of their training. The scenario included history taking and physical examination of a standardized patient. This was a hybrid simulation; a synthetic pelvic task trainer that allowed the placement of the cervical model was connected to the standardized patient. The task trainer was placed under a drape and appeared to extend from the standardized patient’s body. At various points in the simulation, the standardized patient controlled the cervical bleeding through a peripheral venous line. Feedback forms were completed, and the models were discussed and evaluated with staff.

**Results:**

A final cervical model was created and successfully printed. Overall, the models were reported to be similar to a real cervix. The models bled well. Most models were not sutured during the scenarios, but overall, the value of the printed cervical models was reported to be high.

**Discussion:**

The models were well received, but it was suggested that more colors be integrated into the cervix in order to better emphasize the intended pathology. The model design requires further improvement, such as the addition of a locking mechanism, in order to ensure that the cervix stays inside the task trainer throughout the simulation. Adjustments to the simulated blood product would allow the bleeding to flow more vigorously. Additionally, a different simulation scenario might be more suitable to explore the residents’ ability to suture the cervical models, as cervical suturing of a neoplasm is not a common emergency department procedure.

**Conclusion:**

3D-printed cervical models are an economical and anatomically accurate option for simulation training and other educational purposes.

## Introduction

The use of three-dimensional (3D) printing or rapid prototyping in medical simulation training is rapidly evolving [1]. 3D printed simulation has been studied in neurosurgery [2-11], plastic and reconstructive surgery [12-14], and cardiovascular surgery [15-16]. However, little research supports the use of this technology in gynecological simulation. We created a 3D-printed cervix and cervical neoplasm for use in resident-level gynecological simulation training. The style of 3D printing used in this study was fused deposition modeling (FDM), which involves laying down material along the X and Y axis, and then the Z axis, until object completion [17].

**Figure 1:**
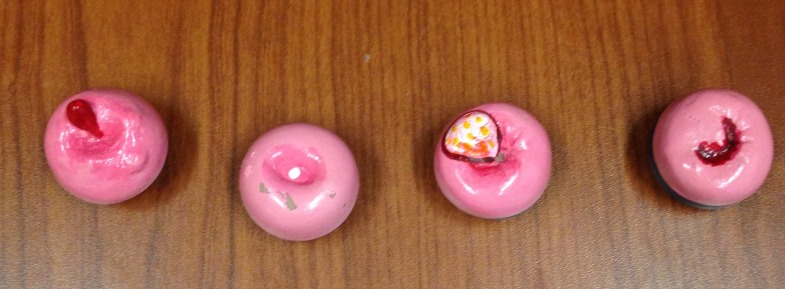
Array of historical cervix models

The department of obstetrics and gynecology has used a hybrid simulation to evaluate residents’ abilities to recognize and manage various gynecological emergencies. For the purpose of the current simulation, a cervical model simulates a bleeding cervical cancer. The primary limitations of current cervical models (Figure 1) in the simulation setting is that unlike a real cervix, they are extremely hard and do not have the capacity to bleed or be sutured. To address these limitations, the department collaborated with the Memorial University of Newfoundland (MUN MED) 3D team to develop a 3D-printed design and model that would address these limitations. Our study aim was to create a low-cost model that looked and felt realistic.

## Materials and methods

The first iteration of the design for the 3D cervix model used historical cervical models as a starting point for overall design dimensions. From here, the design process was guided by physicians practicing in the department of obstetrics and gynecology. The design work was completed using 3D computer-aided design (CAD) software called SolidWorks (SolidWorks, Waltham, MA, USA). These designs were exported as STereoLithography files (STL), which is a compatible format for most 3D printers. All STL files can be made available for those interested through communication with the corresponding study author.

Each iteration of the cervix model was designed using very simple methods and basic SolidWorks features. The generic shape of the model determined the geometry of the initial sketch in SolidWorks (2015–2016). Some of the model designs used sketch features such as revolve and extruded cut, which helped to ensure uniformity and symmetry in the model.

**Figure 2:**
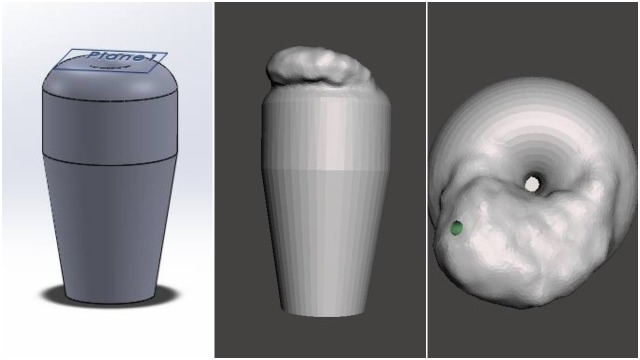
Final design of cervical model in SolidWorks (left) and cervical design with tumor in MeshMixer from a side (center) and aerial view (right)

After the cervix design was felt to be satisfactory, the tumor was added to the base model. The design file was loaded into a free software, MeshMixer (Autodesk, San Rafael, CA, USA), which allowed for the manipulation of triangle meshes constituting the 3D design work. In MeshMixer, the sculpt feature allowed for the creation and customization of a tumor-like mass on the cervix base model. This file was then exported as an STL file for printing. Numerous design alterations were made after prototyping several cervical iterations, which all followed the previously-described process. Additionally, multiple prints were necessary to determine the optimal print material, orientation, and use of support structures. The final 3D design of the cervical model in SolidWorks and the tumor addition in MeshMixer can be viewed in Figure 2.

**Figure 3:**
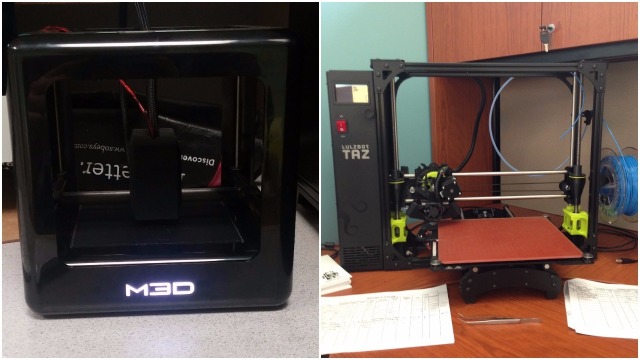
Micro M3D (left) and LulzBot TAZ 6 (right)

The FDM 3D printers used for this study were the M3D Micro (M3D, Fulton, MD, USA) and LulzBot TAZ 6 (Aleph Objects, Loveland, CO, USA) (see Figure 3). The Micro has a 50–350 micron layer resolution, a 15 micron X and Y positioning accuracy, and requires a USB connection. The TAZ 6 uses automatic bed leveling, a heated polyetherimide print bed, an all metal extruder (LulzBot v2 Hot End) (Aleph Objects, Loveland, CO, USA), and a secure digital (SD) card for storing and printing files. The Micro was used to print the early prototypes of the cervix model; however, the TAZ 6 was used to create several functional prototypes, as well as the final version used in the simulation. The range of settings included fill densities, structural supports, bed adhesion tools, and print qualities.

**Figure 4:**
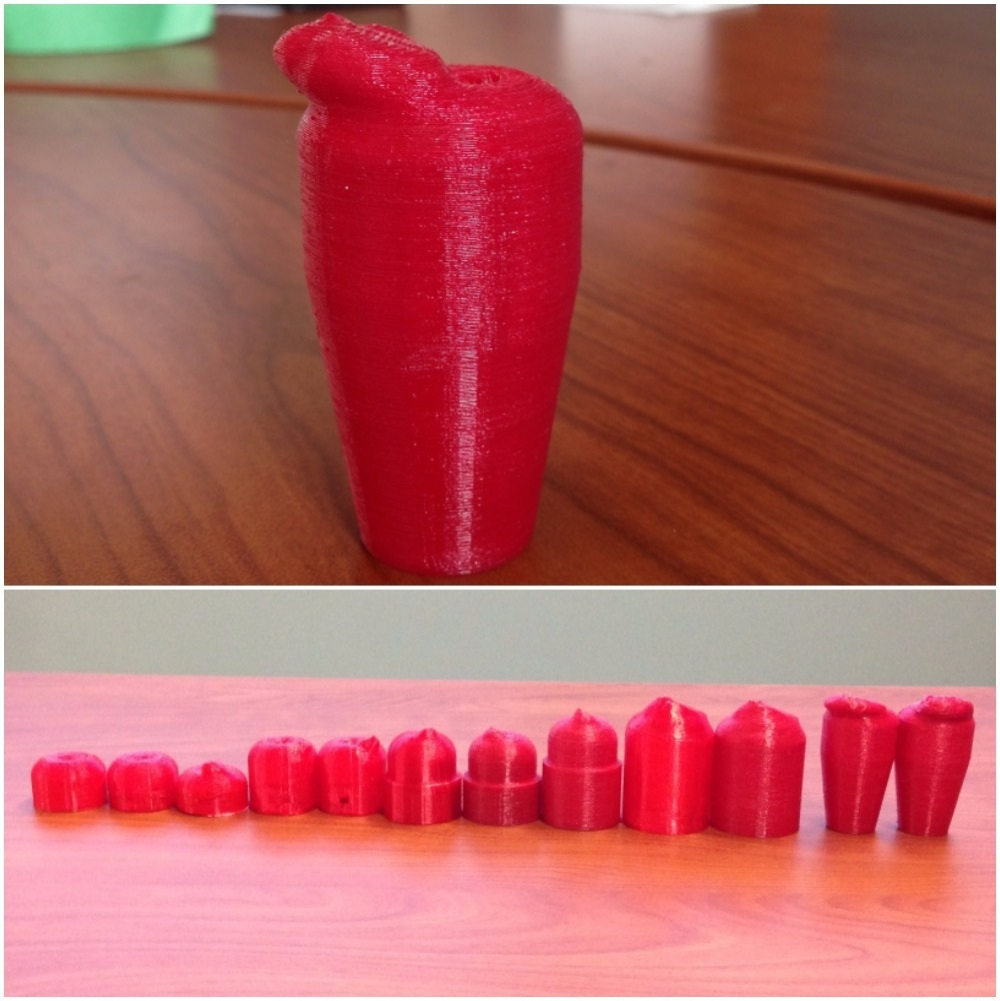
Final cervical model with neoplasm made from SemiFlex with the TAZ 6 (top) and evolution of 3D-printed cervical models with PLA, FLX and SemiFlex materials (bottom)

Additionally, a variety of different filaments were used to create the cervix models. Polylactic acid (PLA) was used to create two of the models at the start of the process. This material is rigid and hard and was simply used to appreciate the overall dimensions of the design. The next material utilized was called Tough 3D Ink (FLX) and is produced by M3D. This material was much more flexible than the PLA, which allowed for the creation of a more realistic model. The final material utilized was called SemiFlex (NinjaTek, Manheim, PA, USA), which was the most suitable material for our study in terms of anatomical material realism. The diameter of the PLA and SemiFlex was 2.85 mm and that of the FLX was 1.75 mm. The PLA and SemiFlex models were printed using the TAZ 6, and the FLX models were only printed using the Micro. The SemiFlex material was the most similar to a human cervix as it was appropriately colored, more flexible, and could tear and be sutured. The final cervix model (Figure 4) was made of this material and featured a hardened neoplasmic mass with a softer cervix surface. A small opening was placed in the middle of the neoplasm as well as a larger hole in the external cervical os. During the simulation, intravenous tubing was connected to the base of the model, which allowed the standardized patient to "control the flow" of the simulated blood, thus creating the appearance of hemorrhaging through the cervical os and neoplasm. The evolution of the various 3D printed models can also be viewed in Figure 4.

The average time taken for printing the cervical model on the Micro was three hours 27 minutes, and the material used was 16.25 g. The average print time for the TAZ 6 was two hours 20 minutes, and the average material used was 23.1 g. After model design modifications were made following discussions amongst the research team, the final model was selected for use in simulation. There were four separate groups of residents; therefore, five cervical models were needed (one for a backup). All five of these models were printed successfully at the same time on the TAZ 6 with a total print time of six hours and 38 minutes and with 55 g of material used, at a total material cost of $8.80 CAN. The amount of material used varied greatly depending on the type of material and printer utilized. Overall, the average cost of each print was $1.26, whereas the average print cost for each material (FLX, PLA-I, SemiFlex) was $1.01, $0.53, and $1.76, respectively. The final model that was used in the simulation cost $1.76 to produce. Information regarding the time, cost, and materials used for each iteration of the design can be observed in Table 1.

**Table 1:** A summary of the material type, amount, and cost for each cervical prototype

Model iteration (from first prototype to final model used for simulation)	Amount of material (meter/grams)	Print time	Material used	Estimated cost ($)
Cervix	1.28/10	46 mins	PLA-I	0.50
Cervix	3.01/8.88	2 hrs 11 mins	FLX	0.55
Cervix w/ cancer	1.29/11	46 mins	PLA-I	0.55
Cervix hollow	2.85/ 8.42	2 hrs 6 mins	FLX	0.52
Cervix sleeve	6.22/18.36	3 hrs 41 mins	FLX	1.14
Cervix sleeve	2.58/21	1 hr 50 mins	SemiFlex	1.37
Cervix sleeve_1	3.38/27	2 hr 9 mins	SemiFlex	1.76
Cervix sleeve large abscess	9.93/29.33	5 hrs 52 mins	FLX	1.82
Cervix sleeve large abscess	4.51/37	2 hr 35 mins	SemiFlex	2.41
Cervix tapered with cancer	2.87/23	1 hr 47 mins	SemiFlex	1.50
Cervix tapered with hollow cancer rev. 1	3.30/27	1 hr 58 mins	SemiFlex	1.76

**Figure 5:**
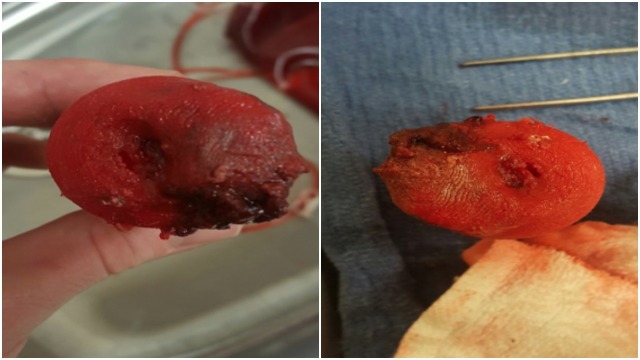
Cervical models with simulation makeup used on resident training day

The 3D-printed cervical model was brought to the simulation center, where makeup and simulated blood products were applied (Figure 5).

**Figure 6:**
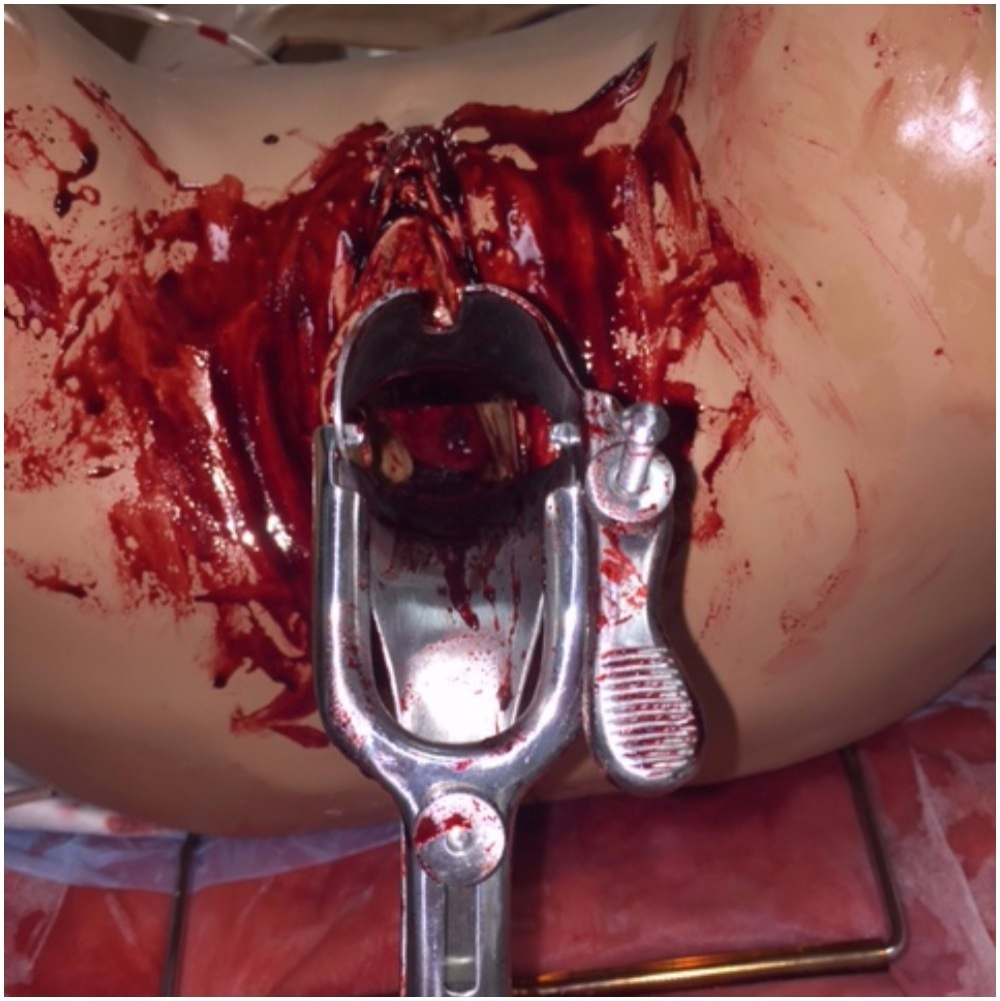
A view of the 3D-printed cervical model inside the task trainer This is an image of the task trainer and cervical model used right before the simulation. Simulated blood was applied by experts in the simulation department and by staff from obstetrics and gynecology who have designed the simulation.

The 3D-printed cervix was inserted into a task trainer, which was connected to the standardized patient (Figure 6). The simulation started with history taking from the standardized patient, which triggered the need for a physical examination. During the physical examination, there was evidence of bleeding coming from the vagina. This prompted the residents to perform a pelvic examination. When using the speculum, the residents were able to visualize the 3D-printed cervical model, and dependent on clinical judgment, an attempt to suture the tissue was prompted. Each group simulation session lasted 30 minutes, after which the groups attended a debriefing session to discuss the simulation. 

**Figure 7:**
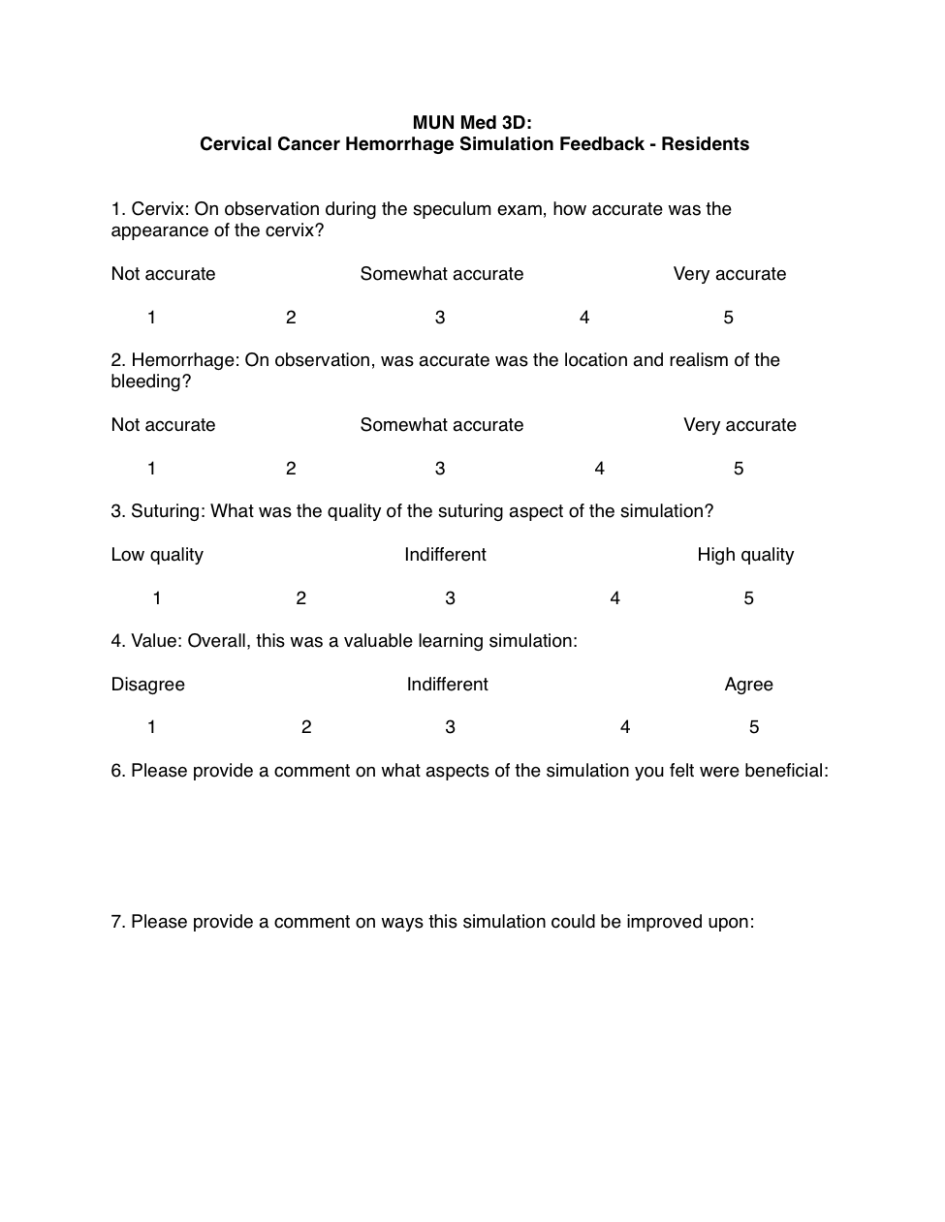
Mean feedback responses to the Likert questions from obstetrics and gynecology residents

At the debriefing session, after informed verbal consent was acquired, nine residents participated in a brief survey evaluating their experience using the cervical models (Figure 7). The simulation staff leads provided verbal feedback regarding the use of the cervical models.

Informed verbal consent was obtained from participants and no identifying information was collected.

## Results

The feedback received from the residents’ responses was positive and supported the use of the 3D-printed cervix for simulation training. The responses from the feedback cannot be used to make definite conclusions due to the low number of study participants, but they may help guide further improvements in follow-up work.

A series of closed-end, 5-point Likert scale questions were asked in the feedback form (Figure 7). Nine feedback forms were completed by the residents, and their mean response rates can be found in Table 2. Respondents noted that the 3D model accurately replicates the appearance of a human cervix. Simulated hemorrhaging was noted to be somewhat accurate, and suturing was attempted only by a single participant (who rated it highly). Overall, resident responses report that the simulation was a valuable learning experience.

**Table 2:** Mean feedback responses to the Likert questions from obstetrics and gynecology residents Although questions surrounding suturing were asked in the feedback form, they have not been included in this table as only one participant responded to this question. Also, one participant did not fill out a response for the question on hemorrhaging, which was reflected in the mean calculation (n=8).

Feedback question	Mean response rate (5 = highest agreeable score)
Cervix: On observation during the speculum exam, how accurate was the appearance of the cervix?	3.6
Hemorrhage: On observation, how accurate was the location and realism of the bleeding?	3
Overall, this was a valuable learning simulation	4.1

Two open-ended questions were asked in the survey (Figure 7): “Please provide a comment on what aspects of the simulation you felt were beneficial” and “Please provide a comment on ways this simulation could be improved upon.” Some highlighted responses include: “the cervix needing to be further stabilized to the task trainer,” “use of multi-colors would be helpful,” and “there was an overall appreciation for the simulation and teaching.”

Feedback from the staff involved in the simulation included the suggestion that the models resembled a human cervix with neoplasm, but could be improved through the use of other colors. Adaptions were made to improve the function of the model throughout the simulation. For example, in the first scenario, the cervical model was inadvertently dislodged into the pelvic model. This was remedied through the use of a cable tie to secure the model. Also, the initial thought was to rotate through a new cervical model with every group; however, the facilitators decided to use only one of the models for all the simulations. The model did withstand all four simulations, but could need to be changed if more suturing was performed amongst the groups. To improve the bleeding realism in the future, the catheter that produces simulated blood may need to be placed above the cervical model.

## Discussion

It is evident that the models were useful for the residents' training; however, there were areas identified for further improvement. Many of the issues encountered were with the model setup and not with the actual 3D model. Some areas for future improvement will be discussed in this section.

It was noted that the use of a red model was helpful; however, a variety of colors exist in an actual patient’s anatomy. These findings were supported by feedback from the residents and staff. We have begun looking at the possibility of adding a second extruder to the printer, which would allow for future models to be printed with multiple colors and materials. Both multicolor and material models will improve the visualization of the tumor and may better mimic specific tissue pathologies in future models.

The model design may be altered. As noted in the responses to the question on hemorrhage, the blood did not flow through the tumor as one might expect in an actual patient. The design may be changed in order to connect the tubing directly to the tumor, instead of in a cavity where the blood expels through the os and tumor. The opening of the os can also be reduced. Additionally, in the first simulation group, the speculum pushed the cervix through the back of the trainer. This was fixed by using a cable tie around the base of the cervix to secure it in the task trainer. In the future, the base of the cervical model can be directly anchored to the trainer with a hook.

The models received minimal suturing during the simulation. However, it was reported that it would have been useful to suture the models. Residents' level of experience or incomplete instruction provided during the simulation may contribute to reasons why suturing was not attempted. This prototype study has not collected adequate data to address this issue. The simulation setting (emergency department consult) itself may not be ideal for suturing, as residents noted that the suturing of a neoplasm would not occur there. Additionally, the cervix was dislodged in one of the sessions. This may have prevented the trainees from suturing the model. It is important to know if the material and techniques used for printing the models were sufficient to enable suturing. In follow-up work, we will ensure that the residents are clearly instructed to attempt suturing the models and will evaluate this process. Other potential scenarios are being explored that may better accommodate suturing.

## Conclusions

A more accurate and realistic cervical model can be developed using 3D printing. The cost of design and production is minimal. The material used for the final cervical model cost $1.76 CAN. Additionally, pathology may be added. Simulations may be modified to suit the trainees' level of experience. Multiple colors and materials may enhance low-fidelity models. The field of gynecology may benefit from further innovation with 3D printing. This can hopefully lead to improved, low-cost, and individualized training.
